# Volume of Lateral Geniculate Nucleus in Patients with Glaucoma in 7Tesla MRI

**DOI:** 10.3390/jcm9082382

**Published:** 2020-07-26

**Authors:** Ewa Kosior-Jarecka, Anna Pankowska, Piotr Polit, Andrzej Stępniewski, Mark Roger Symms, Paulina Kozioł, Tomasz Żarnowski, Radosław Pietura

**Affiliations:** 1 Department of Diagnostics and Microsurgery of Glaucoma, Medical University of Lublin, 20-079 Lublin, Poland; piotrjanpolit@gmail.com (P.P.); zarnowskit@poczta.onet.pl (T.Ż.); 2 Department of Radiography, Medical University of Lublin, 20-079 Lublin, Poland; zubianna@gmail.com (A.P.); paulina.koziol@op.pl (P.K.); radoslawpietura@gmail.com (R.P.); 3 Centrum ECO-TECH COMPLEX Maria Curie-Skłodowska University in Lublin, 20-612 Lublin, Poland; astep@ipan.lublin.pl; 4 Applied Science Labs, GE Healthcare, Amersham HP7 9NA, UK; MarkRoger.Symms@ge.com

**Keywords:** glaucoma, 7Tesla MRI, lateral geniculate nucleus

## Abstract

The aim of the study was to assess the volume of the lateral geniculate nucleus (LGN) in patients with open-angle glaucoma in 7Tesla MRI and to evaluate its relation to RNFL thickness and VF indices. Material and methods. The studied group consisted of 20 open-angle glaucoma patients with bilaterally the same stage of glaucoma (11 with early glaucoma and nine with advanced glaucoma) and nine healthy volunteers from the Department of Diagnostics and Microsurgery of Glaucoma, Medical University of Lublin, Poland. Circumpapillary RNFL-thickness measurements were performed using OCT in all patients and visual fields were performed in the glaucoma group. A 7Tesla MRI was performed to assess the volume of both lateral geniculate bodies. Results. The LGN volume varied significantly between groups from 122.1 ± 14.4 mm^3^ (right LGN) and 101.6 ± 13.3 mm^3^ (left LGN) in the control group to 80.2 ± 17.7 mm^3^ (right LGN) and 71.8 ± 14.2 mm^3^ (left LGN) in the advanced glaucoma group (right LGN *p* = 0.003, left LGN *p* = 0.018). However, volume values from early glaucoma: right LGN = 120.2 ± 26.5 mm^3^ and left LGN = 103.2 ± 28.0 mm^3^ differed significantly only from values from the advanced group (right LGN *p* = 0.006, left LGN *p* = 0.012), but not from controls (right LGN *p* = 0.998, left LGN *p* = 0.986). There were no significant correlations between visual field indices (MD (mean deviation) and VFI (visual field index)) and LGN volumes in both glaucoma groups. Significant correlations between mean RNFL (retinal nerve fiber layers) thickness and corresponding and contralateral LGN were observed for the control group (corresponding LGN: *p* = 0.064; contralateral LGN: *p* = 0.031) and early glaucoma (corresponding LGN: *p* = 0.017; contralateral LGN: *p* = 0.008), but not advanced glaucoma (corresponding LGN: *p* = 0.496; contralateral LGN: *p* = 0.258). Conclusions. The LGN volume decreases in the course of glaucoma. These changes are correlated with RNFL thickness in early stages of glaucoma and are not correlated with visual field indices.

## 1. Introduction

Glaucoma—a multifactorial disease involving retinal ganglion cells (RGC)—has been estimated as the second leading cause of blindness throughout the world [1]. Pathologically, dendritic damage, cell body shrinkage and death are ultimately manifested as a progressive loss of the RGC population, leading to clinical vision loss [2,3,4]. An increasing number of studies [2,3,4,5] have suggested that primary open-angle glaucoma (POAG) causes changes in the whole visual pathway, including the optic nerve, lateral geniculate body (LGN), optic radiation and visual cortex. Moreover, the early morphologic changes were detected in the central nervous system (CNS) in monkeys [4].

Multicenter studies showed that elevated intraocular pressure (IOP) is the major risk factor for the onset and progression of glaucoma and the only modifiable factor in treatment. However, some patients continue to progress despite the reduction in IOP levels [6], while others develop typical glaucomatous damage in the absence of elevated IOP (normal tension glaucoma). These data confirm that the pathogenic mechanisms underlying glaucoma are complex which shows the need to look for novel therapeutic strategies.

The lateral geniculate nucleus (LGN) plays a role in the function of perception and cognition, including visual attention and awareness [7]. Most RGCs in the LGN form the major vision center relaying information from the eye to the visual cortex. In the LGN the complete contralateral hemifield of vision is represented and parallel central visual pathways are segregated into anatomically distinct magnocellular, parvocellular and koniocellular channels. The axons of the RGCs associated with the temporal half of the retina do not cross in the optic chiasm and transmit their signal to the ipsilateral LGN. In contrast, the RGC axons from the nasal half of the retina decussate in the optic chiasm and transmit the signal to the contralateral LGN. The axons of LGN neurons form the optic radiations projecting to eye-specific columns in the primary visual cortex [2].

In this study, we aimed to include the patients with the same stage of glaucomatous neuropathy in both eyes assessed according to visual field parameters, something which has never been taken into consideration in previous studies. We hoped that this approach would diminish the doubts caused by the fact that the part of nerve fibers crosses the optic nerve in the chiasm, so different glaucoma stages in both eyes theoretically affect the two LGN unequally.

Glaucomatous visual losses can be prevented with early diagnosis and treatment; however, affected individuals are typically asymptomatic until the advanced stages. Additionally, although standard automated perimetry (SAP) is the gold standard test of visual function in glaucoma, histological studies have indicated that large numbers of RGC are lost before statistically significant visual field abnormalities [8]. Therefore, there is still a need to look for more sensitive and specific diagnostics methods to be used in glaucoma.

Magnetic resonance imaging (MRI) has been used to analyze the visual pathway in glaucoma in previous studies; including morphologic examinations [9,10] and functional measurements [11]. MRI of high resolution with morphometric assessment of LGN and further visual pathway may give the valuable structural diagnostic option in glaucoma, supplementing the typically used structural measurements of retinal nerve fiber layer and optic disc and functional examination by visual field. The assessment of the utility of MRI techniques in glaucoma diagnosis needs to be further studied.

Li [12] showed that fractional anisotropy–an index derived from diffusion tensor imaging–can be a measure of glaucoma damage in the optic tract. Lee et al. [13] performed volumetric measures of the LGN in 7T MR with proton density weighting. The higher signal-noise-ratio at 7T allows the acquisition of images with increased spatial resolution [14]. This facilitates precise and possibly better delineation of the LGN based on high-resolution anatomic datasets when compared to standard MR imaging at 3T.

The aim of the study was to assess the LGN volumes in patients with bilaterally the same stage of open-angle glaucoma in 7Tesla MRI and to evaluate its relation to RNFL thickness and VF indices.

We acquired 7T MR images using Silent [15]–a three-dimensional version of RUFIS [16]–with magnetization transfer weighting. The LGN can be clearly seen on these images, facilitating accurate volumetric study.

## 2. Material and Methods

The study design was approved by the local Ethical Committee Board at the Medical University of Lublin (approval number KE 0254/149/2018). Informed consent was signed by all participants before inclusion and the study was performed in accordance with the tenets of the Declaration of Helsinki.

The studied group consisted of 20 open-angle glaucoma patients and 9 age- and sex-matched healthy volunteers from the Department of Diagnostics and Microsurgery of Glaucoma, Medical University of Lublin, Poland.

All studied patients underwent ophthalmic examinations: best-corrected visual acuity (BCVA) assessment, slit-lamp biomicroscopy, Goldmann applanation tonometry, gonioscopy, central corneal thickness and clinical stereoscopic optic disc evaluation. Additionally, visual field examination (perimetry) and optical coherent tomography scanning (OCT) were performed. Standard Automated Perimetry (SAP) was performed using 30 ± 2 Swedish Interactive Threshold Algorithms (Humphrey Field Analyzer II, Carl Zeiss Meditec, Dublin, OH, USA). Global indices, such as mean deviation (MD) and Visual Field Index (VFI), of the last reliable SAP were chosen for statistical analysis. Peripapillary retinal nerve fiber layers (RNFL) were measured using OCT (Stratus, Carl Zeiss Meditec, Dublin, OH, USA). The neuroretinal rim area was measured in 4 sectors (temporal, superior, nasal, inferior) and globally as mean RNFL thickness.

Glaucoma was defined clinically as glaucomatous optic nerve head morphology combined with typical, reproducible defects in SAP. The glaucomatous defect on SAP was defined based upon a glaucoma hemifield test result qualified as outside normal limits and with the presence of at least 3 contiguous test points within the same hemifield on the pattern deviation plot at *p <* 1%, with at least 1 point at *p <* 0.5%, on at least 2 consecutive tests, with reliability indices better than 15%. Open angle was evaluated in gonioscopy according to Schaffer’s criteria with grades III or IV.

The patients needed to meet the inclusion criteria as follows:

- Open angle glaucoma diagnosed according to the criteria mentioned above,
- Bilaterally the same glaucoma stage. Glaucoma staging was evaluated according to Hodapp–Parish–Anderson classification of visual field loss. Subjects with mean deviation (MD) in each eye better than −6 dB were classified as the early glaucoma group (11 patients) and subjects with MD in each eye worse than −12 dB patients were classified as the advanced glaucoma group (9 patients).

Healthy volunteers underwent ophthalmic examination. The control group consisted of subjects with IOP *<* 21 mmHg in both eyes, normal optic disc appearance in ophthalmoscopy, RNFL values within normal limits, and no ocular or systemic abnormalities that could affect the optic nerve structure or visual function.

Imaging data were obtained at the Ecotech Complex (Lublin, Poland) using a Discovery MR950 7T MRI system (7T23_v03 software) (GE Healthcare, Chicago, IL, USA) with a gradient strength of 50 mT/m and a slew rate of 200 T/m/s. The coil configuration used for examinations was a two-channel birdcage coil driven in quadrature for transmission and a 32-channel array coil for reception (Nova Head 32-channel head coil, 2Tx/32Rx). All patients who participated in the study were previously examined in a 1.5 T scanner (Optima360, GE Healthcare, Chicago, Illinois, USA). They had routine brain and orbits examination to exclude the presence of pathologies of the central nervous system or any other pathology which could confound the glaucoma diagnosis. The imaging protocol in the 7-T examination contained four sequences: 3D BRAVO T_1_, FSE XL T_2_, 3D CUBE T_2_, and 3D MT-weighted Silent (obtained with parameters from Table 1). The 3D sequences (BRAVO, CUBE and MT-Silent) were all of high isotropic resolution (1 mm or smaller) to enable precise segmentation. The FSL–XL sequence was included as a radiological reference. All sequences had minimal geometric distortion in the region studied. For LGN volume evaluation the 3D MT-weighted Silent sequence was used.

**Figure 1:**
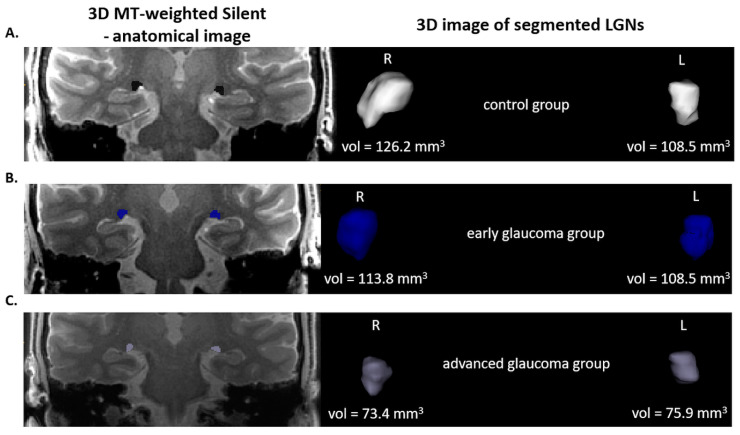
On the left side—3D MT-weighted Silent—the anatomic image of the brain with marked LGNs. Right—results of LGNs manual segmentation. Images show one case chosen from each studied group. (**A**) control group; (**B**) early and (**C**) advanced glaucoma. Images acquired at the Ecotech Complex (Lublin, Poland).

The 3D MT-Silent sequence was evaluated independently by two radiologists (AP, RP). The radiologists were not aware of the clinical status of the examined patients at the time of evaluation. Manual segmentation and volume calculation of LGNs and thalamic were performed using ITK-SNAP software (version 3.8; PA, USA) [17]. In case of LGN volumes the presented results are the mean values of two attempts made by each radiologist whereas in the case of the thalamus each researcher made only one attempt (results in Table 4). Moreover, cross-investigator comparisons of LGNs volume were performed using Friedmann’s ANOVA nonparametric test and Kendall’s coefficient of concordance ($\tilde{W}$) to exclude possible discrepancies between two researchers (results in Table 3). Example images of LGNs in the 3D MT-Silent sequence are presented in Figure 1.

All statistical analyses were performed in Statistica 13 (StatSoft, Inc., Tulsa, OK, USA). The normality of distribution of the variables was assessed by the Shapiro–Wilk test. LGN volume comparison was made using one-way ANOVA (post hoc Tukey’s test for data that met the conditions of normal distribution and nonparametric Kruskal–Wallis test for the remaining data). Correlation analysis shown was performed using the Pearson’s and Spearman’s correlation tests. All results were considered significant at *p* < 0.05.

## 3. Results

Table 2 indicates that there are no significant differences between the age of patients in the examined groups. Comparison of the visual field MD values of both eyes reveals there is a significant decrease of values between groups ranging from 0.36 dB (right eye, control group) to −19.70 dB (left eye, advanced glaucoma group). Furthermore, the VFI of both eyes differs significantly between control and early glaucoma groups (VFI right, *p* = 0.034; VFI left, *p* = 0.014) and between control and advanced glaucoma groups (VFI right, *p* = 0.00001; VFI left, *p* = 0.00006). Furthermore, RNFL thickness shows a significantly decreased score between control and advanced groups (right eye *p* = 0.0005, left eye *p* = 0.0006). Left RNFL thickness of the control group compared with the early glaucoma group also reveals a significant dependence (*p* = 0.018). All differences between clinical parameters were analyzed using one-way ANOVA statistical analysis (post hoc Tukey’s test).

**Figure 2:**
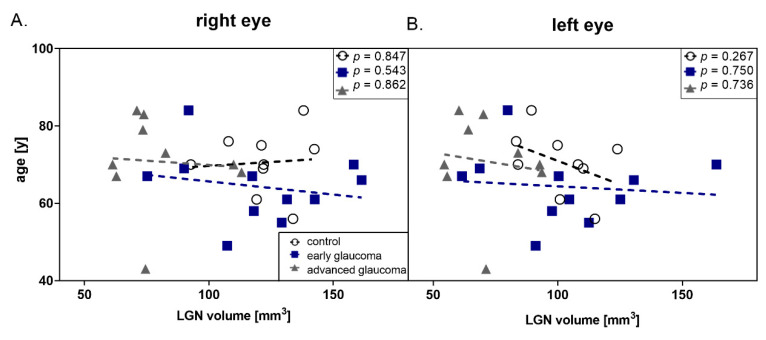
Correlation between age and lateral geniculate nucleus (LGN) volume in all groups (black dot—control; dark blue square—early glaucoma, gray triangle—advanced glaucoma) (**A**) right eye (**B**) left eye.

Figure 2 shows the correlations of LGN volume and age of patients in all examined groups. None of the dependencies presented in this figure proved to be statistically significant (values presented in Table 5).

Considering the possibility of the discrepancy of LGNs manual segmentation results between the two attempts of two investigators, a concordance assessment was performed. The analysis revealed high concordance rates (presented in Table 3) during volume analysis in all groups, both of the right and left LGNs. Kendall’s coefficient of concordance calculated according to Friedmann’s ANOVA ranged from 0.69 (right LGN volumes of advanced glaucoma group, *p* = 0.005) to 0.94 (right LGN volumes of early glaucoma group, *p* = 0.00005).

**Figure 3:**
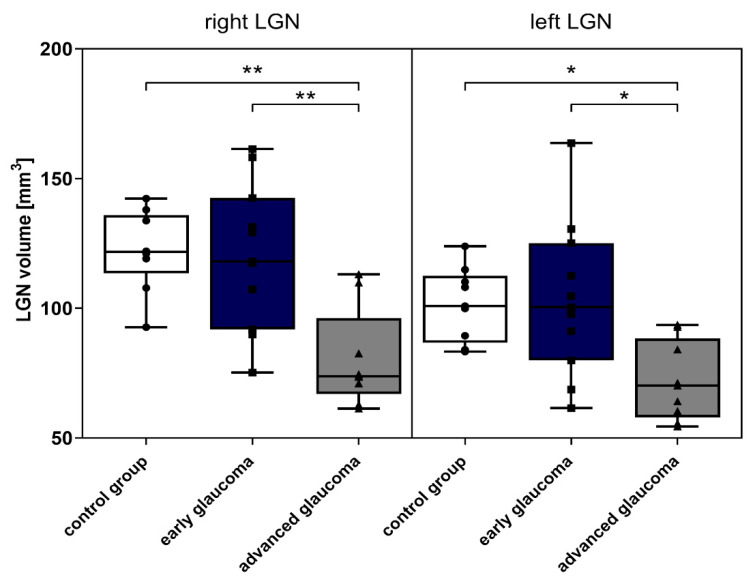
LGNs volume in examined groups—control, early and advanced glaucoma. Data represent Table 1. * *p* < 0.05, ** *p* < 0.01).

Considering the average values presented in Table 3 and Table 4 there were no significant differences between LGNs volume of controls (right LGN = 122.1 mm^3^, left LGN = 101.6 mm^3^) and the early glaucoma group (right LGN = 120.2 mm^3^, left LGN = 103.2 mm^3^) (*p* = 0.998 and *p* = 0.986, right and left LGN, respectively). However, there were significant differences when comparing the advanced glaucoma group (right LGN = 80.2 mm^3^, left LGN = 71.8 mm^3^) to the early glaucoma group (right LGN *p* = 0.006, left LGN *p* = 0.012) and to healthy controls (right LGN *p* = 0.003, left LGN *p* = 0.018), as shown in Figure 3.

Table 4 presents the results of the manual segmentation of anatomic structures (LGN and thalamus) using a 3D MT-weighted Silent sequence. The volume of right and left thalamus in the control group revealed decreased values (right thalamus = 5326 mm^3^, left thalamus = 5152 mm^3^) in comparison to glaucoma groups (early: right thalamus = 5842 mm^3^, left thalamus = 5697 mm^3^; advanced: right thalamus = 5792 mm^3^, left thalamus = 5696 mm^3^). However, the results do not differ significantly.

Moreover, Table 4 indicates that the intragroup results of volume of LGN and thalamus do not differ between right and left side (except the volume of LGN in the control group, *p* = 0.009).

**Figure 4:**
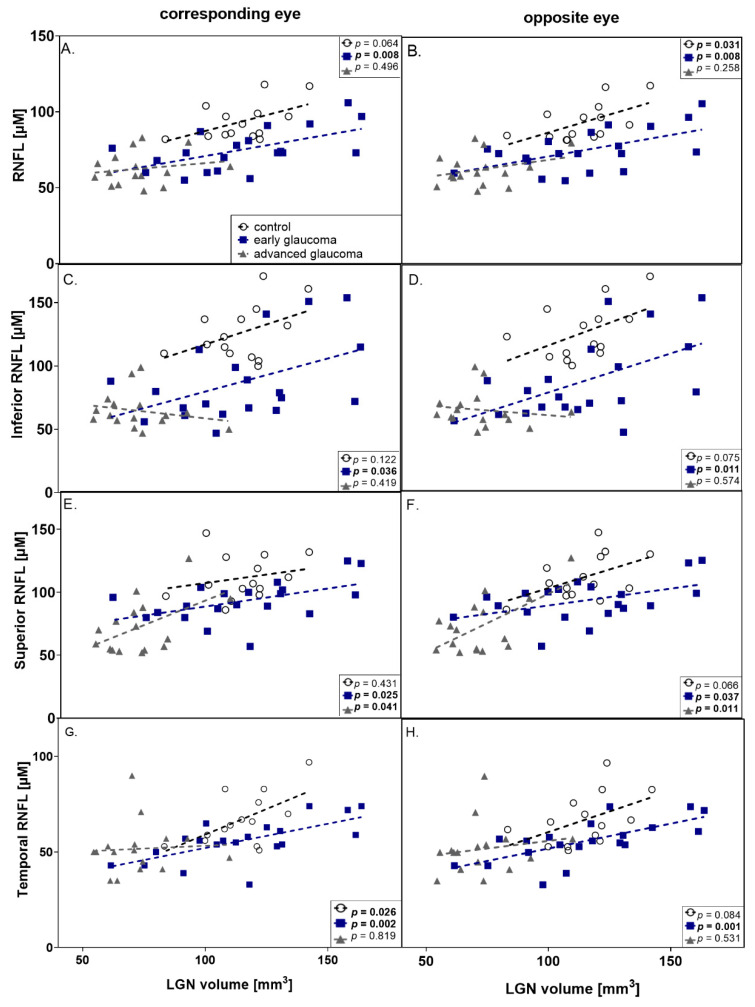
Graphic presentation of correlations between RNFL thickness and LGNs volume in corresponding (left column) and opposite eye (right column). (**A**,**B**) mean RNFL thickness; (**C**,**D**) inferior RNFL thickness; (**E**,**F**) superior RNFL thickness; (**G**,**H**) temporal RNFL thickness; all are correlated with LGN volume. Correlation coefficients of Pearson’s (r) and Spearman’s (R) are presented in Table 5. (black dot–control group, dark blue square—early glaucoma, gray triangle—advanced glaucoma).

Parametric (Pearson’s) and nonparametric (Spearman’s) correlations analysis (presented in Table 5 and graphically on Figure 4) based on clinical data and LGNs volume showed that there were no significant correlations between visual field indices (MD and VFI) and LGN volumes in both glaucoma groups. However, the correlations between mean RNFL thickness of contralateral LGNs were observed for the control group (*r* = 0.58, *p* = 0.031; *R* = 0.58, *p* = 0.031) and for early glaucoma (*r* = 0.58, *p* = 0.008; *R* = 0.53, *p* = 0.016), but not advanced glaucoma. Referring to RNFL thickness and corresponding LGNs volume there is a significant correlation only in the early glaucoma group (*r* = 0.57, *p* = 0.008; *R* = 0.53, *p* = 0.017). Table 5 and Figure 4 presents the details of the correlation of LGN volumes with RNFL thickness in quadrants (inferior, superior, nasal and temporal). The most statistically significant correlations occur in the temporal quadrant (control and early glaucoma groups), while no significant correlations are observed in the nasal quadrant.

## 4. Discussion

The high isotropic resolution (<0.8 mm) and excellent gray/white matter contrast of MT-Silent facilitate the depiction of small structures in the brain such as the LGN. The magnetization transfer contrast is generated by the Fat Suppression pulse [18]. Magnetization Transfer Ratio imaging has been shown to be sensitive to pathologic changes caused by stroke [19,20], optic neuritis [21] and Multiple Sclerosis [22]. The cause of these changes is often attributed to axonal loss and demyelination [23], though a lack of complete confirmation by pathology means this explanation is not universally accepted [24]. Our data does not include an image acquired without magnetization transfer weighting, which is necessary to calculate the MT ratio, so we cannot make similar inferences about MTR for our subjects. This limits our use of MT-Silent in this study to segmentation (measuring volumes), rather than quantitation (measuring signal intensities).

Glaucoma degeneration is not just present in the RGCs. Two possible pathogenic mechanisms underlying glaucomatous neuropathy may be hypothesized. One suggests that glaucoma begins with RGCs loss and leads to the transmission of termination signals to the LGN and then to the visual cortex. However, the other possibility is that the LGN may at least in some cases be the primary site of damage leading to a reduction of the axonal transport of neurotrophic factors, given that RGCs are more susceptible to local injuring factors [25]. Additionally, lesions of the LGN or primary visual cortex may induce RGC degeneration, through a decrease in trophic support [26]. Experimental data showed that elevated IOP caused loss of both RGCs and LGN neurons and the neuronal degeneration proceeded from the retina to the LGN [27,28]. There were also studies showing that glaucoma may be a manifestation of neurodegeneration in the central nervous system [29]. Other evidence indicates a link between glaucoma and Alzheimer’s disease [30,31]. Moreover, the loss of specific neuronal populations, the induction of mechanisms of cell injury and the deposition of protein aggregates in specific anatomic areas are very similar events among Alzheimer’s and Parkinson’s disease and glaucoma [32]. In this study, when comparing healthy controls with the glaucoma group, a significant difference in mean RNFL thickness was observed, although there were no differences in LGN volumes. This shows that the RNFL thickness tended to be a more sensitive parameter for differentiation of healthy subjects from those with early glaucoma. However, strong correlations between mean RNFL thickness, superior, inferior, temporal RNFL thickness, and the LGN volume were shown. These anatomic findings conflict with the theory that the glaucomatous process starts in the LGN. On the other hand, in our study, patients with high tension and normal tension open-angle glaucoma were not differentiated.

The partial crossing of optic nerves in the chiasm impedes the assessment of the glaucoma impact on LGN volume. In this study, we decided to include only patients with bilaterally the same stage of glaucomatous neuropathy assessed according to visual field results to avoid the bias caused by partial crossing of the optic nerve in the chiasm. The lack of differences between the volume of right and left LGN in patients with early and advance glaucoma confirms the same bilateral glaucomatous stage.

There is only limited data on staging glaucoma according to the structural damage [33]. Evaluation of glaucoma stage according to VF results plays a crucial role in clinical practice, but it does not always reflect the structural changes: in early glaucoma, the changes in RNFL thickness better correlate with the course of glaucoma. However, the use of the Hodapp–Parish–Anderson classification of visual field loss may also be a limitation of this study, because we did not observe a correlation between MD and LGN volume.

We observed significant differences in LGN volume between advanced glaucoma groups compared to both controls and early glaucoma patients, whereas no significant difference was observed between early glaucoma and controls. To exclude the possible impact of factors unrelated to glaucoma on LGN volume, we decided to compare the volume of thalamic nucleus, as this structure is not connected with visual pathway; no significant difference between the studied groups was observed. Additionally, although age causes axons to lose [32], in this study we did not observe the correlations between LGN volume and age.

Our results partially confirm a previous study using high-resolution 7.0-T MRI, in which the LGN volumes were identified to be significantly smaller in POAG patients, as compared to healthy subjects [13]. In the study by Furlanetto [34] of 3T MRI of LGN, however, the only parameter that showed a significant difference between glaucoma and control groups was the LGN height. Furthermore, at 3T Li [12] examined 24 POAG patients and 24 controls and concluded that for distinguishing glaucomatous eyes from healthy eyes, measurements of the LGN volume exhibited a high sensitivity and specificity. They also observed that the use of LGN volume differentiates glaucomatous patients from healthy controls only at the advanced stage. On the other hand, we observed the highest variability in LGN volume in the early glaucoma group. It is possible that this early group, when identified by the VF test, is the most heterogeneous because half of the RGC population undergoes apoptosis before the appearance of the VF defect. There are data showing that during the course of glaucoma there are different populations of affected RGC, some of them lacking function before apoptosis [35].

In this study, we observed that the more advanced stages of glaucoma patients tended to have lower volumes of LGN. We decided to look for correlation not only with MD, but also with VFI, which is claimed to correlate better to changes in VF significant to the patients (e.g., more central) and may better correlate with the number of supporting RNFL. Despite the relation between glaucoma staging assessed according to VF results, we could not show the correlation between global visual field indices and LGN parameters for either parameters—MD or VFI. This is in accordance with clinical findings of the lack of the correlation between structure and function in both [36] early and advanced glaucoma. However, in the study by Gupta the reduction in LGN volume correlated with glaucoma stage assessed by VF parameters using the Hodapp–Anderson–Parrish system [3].

Glaucoma is related to excessive apoptosis of retinal ganglion cells. However, POAG remains undetected in 50% of cases mainly due to the lack of specific symptoms. The early stage of the disease, preperimetric glaucoma, is characterized by glaucomatous changes undetectable with visual field examination. At this stage, the diagnosis and measurement of glaucoma progression is possible only by structural techniques, with RNFL measurement being the most accurate. Therefore, the early glaucoma changes are better visualized by RNFL measurements and the more advanced with visual field examination. A similar tendency for glaucoma staging was observed in changes of LGN volume in this study. For the control group and for early glaucoma, LGN volume significantly correlated to mean values of RNFL and such correlation was absent in advanced glaucoma. Histologically, except for RGC axons, non-neuronal elements such as blood vessels and retinal glial cells may be included in the measurement of peripapillary RNFL thickness. Even if all RGCs die during the course of glaucoma, non-neuronal components do not degenerate concurrently. Additionally, Wang et al. [37] demonstrated that glial cell proliferation occurs in glaucomatous retinas and is especially prominent in the peripapillary area. Thus, peripapillary RNFL measurements do not proceed to zero level in glaucoma because of the so-called “floor effect” and this phenomenon may be the reason for the lack of the correlation observed in this study. Theoretically, the LGN volume should be resistant to “floor effect” which would make it a better structural indicator than RNFL thickness in advanced glaucoma, but this hypothesis needs further study.

In this study, in early glaucoma the LGN volume was significantly positively correlated to the contralateral mean RNFL thickness. A similar statistical tendency was observed, correlating LGN volume with mean RNFL thickness for the eye on the same side. These results confirm the study performed by Schmidt et al. for NTG patients [14]. Chen et al. [38] indicated that LGN atrophy was consistent with the damage of the optic disc in POAG patients. However, on the contrary, Lee found no correlation between LGN volume evaluated with 7T MRI and average cpRNFL thickness or optic disc parameters in the POAG group [13]. The differences may be connected with the glaucoma stage of included patients, in this study such correlation also was not present for advanced glaucoma.

This study has some possible limitations. First, patients with different types of open-angle glaucoma were included: primary open angle with high tension, normal tension and pseudoexfoliative glaucoma: possible miscellaneous mechanisms underlying neuropathy may affect the results of the study. The glaucoma staging was assessed according to functional parameters. Additionally, we did not include patients with moderate glaucoma in order to see possible differences in LGN in groups from different ends of the glaucoma spectrum.

To sum up, we observed diminishing LGN volume in the course of glaucoma, but this was significant only in advanced stages; RNFL decrease was observed earlier in the course of glaucoma than changes in LGN volume. The LGN volume in early stages was correlated with RNFL thickness which does not support the theory of earlier central injury in glaucoma. We could not observe the correlation between LGN volume and VF indices and did not show structural–functional correlation in early and advanced glaucoma.

## Figures and Tables

**Table 1:** Parameters of the imaging protocol.

	TR	TE	TI	ETL	Image Resolution	FOV	NEX	Matrix Size
	(ms)	(ms)	(ms)		(mm)	(cm)		
Ax 3D BRAVO T_1_	6.5	2.5	450	1	1 × 1 × 1	22	1	288 × 288
Cor FSE XL T_2_	9230	21.3	–	24	1.4 × 1.4	18	3	384 × 384
Sag 3D CUBE T_2_	2500	72.3	–	100	1 × 1 × 1	24	1	256 × 256
Cor 3D MT-Silent	257	0	–	1	0.75 × 0.75 × 0.75	18	3	224 × 224

Definition of table terms: TR = Repetition Time; TE = Echo Time; TI = Inversion Time; ETL = Echo-Train Length; FOV = Field of View; NEX = Number of Excitations.

**Table 2:** Clinical characteristic of the examined groups (means ± SD)—one-way ANOVA statistical analysis (Kruskal–Wallis and Tukey’s test).

	Control Group	Primary Glaucoma	Advanced Glaucoma	*p*-Level		
	*n* = 9 18 Eyes	*n* = 11 22 Eyes	*n* = 9 18 Eyes	Control vs. Early	Control vs. Advanced	Early vs. Advanced
Age (y)	70.5 ± 7.8	64.3 ± 8.7	70.7 ± 11.5	0.388	0.999	0.363
Visual field MD right (dB)	0.36 ± 0.70	−4.85 ± 3.18	−17.37 ± 4.20	**0.049**	**<0.00001**	**0.020**
Visual field MD left (dB)	−0.04 ± 1.08	−4.34 ± 3.13	−19.70 ± 7.13	0.076	**<0.00001**	**0.021**
RNFL thickness right (µm)	92.8 ± 11.6	75.7 ± 13.8	60.2 74.7 ± 11.0	0.059	**0.0005**	0.071
RNFL thickness left (µm)	94.7 ± 11.9	74.7 ± 13.3	64.7 74.7 ± 9.4	**0.018**	**0.0006**	0.272
VFI right (%)	99.9 ± 0.3	85.6 ± 9.7	51.8 ± 12.9	**0.034**	**0.00001**	0.086
VFI left (%)	99.9 ± 0.3	87.4 ± 13.0	46.3 ± 24.5	**0.014**	**0.00006**	0.259

RNFL: retinal nerve fiber layers; VFI: visual field index.

**Table 3:** LGNs volume evaluation performed by two blinded and independent investigators (two attempts by each investigator) in control, early and advanced glaucoma groups (means ± SD). Concordance assessment based on Friedmann’s ANOVA nonparametric test and Kendall’s coefficient of concordance ($\tilde{W}$) (** *p* < 0.01, *** *p* < 0.001).

		Investigator 1	Investigator 2	Average	Concordance Assessment
		Mean ± SD (mm^3^)	Mean ± SD (mm^3^)	Mean ± SD (mm^3^)	$\mathrm{Kendall}’s\;\tilde{W}$
**Control**					
	**right LGN**	119.9 ± 15.9	124.2 ± 17.2	122.1 ± 14.4	0.70 **
	**left LGN**	96.1 ± 14.3	107.1 ± 15.6	101.6 ± 13.3	0.78 **
**Early**					
	**right LGN**	119.0 ± 30.4	121.4 ± 25.3	120.2 ± 26.5	0.94 ***
	**left LGN**	101.1 ± 30.9	105.4 ± 28.5	103.2 ±28.0	0.84 ***
**Advanced**					
	**right LGN**	78.5 ± 22.0	81.9 ± 15.2	80.2 ± 17.7	0.69 **
	**left LGN**	68.3 ± 18.5	75.3 ± 13.0	71.8 ± 14.2	0.82 **

**Table 4:** LGNs and thalamic volumes within examined groups obtained from manual segmentation of 3D MT-weighted Silent images.

	Control Group	Early Glaucoma	Advanced Glaucoma	Group Comparison (*p*-Level)			Right vs. Left (*p*-Level)		
				Control vs. Early	Control vs. Advanced	Early vs. Advanced	Control	Early	Advanced
**Mean right LGN volume (mm^3^)**	**122.1 ± 14.4**	**120.2 ± 26.5**	80.2 ± 17.7	0.998 0.998	**0.003** **0.009**	**0.006** **0.012**	**0.009** **0.027**	0.180 0.360	0.289 0.289
**Mean left LGN volume (mm^3^)**	101.6 ± 13.3	103.2 ±28.0	71.8 ± 14.2	0.986 0.986	**0.018** **0.036**	**0.012** **0.036**			
**Mean right thalamus volume (mm^3^)**	5326 ± 366	5842 ± 674	5792 ± 505	0.143 0.286	0.120 0.360	0.980 0.980	0.320 0.960	0.667 1.000	0.721 0.721
**Mean left thalamus volume (mm^3^)**	5152 ± 325	5697 ± 653	5696 ± 563	0.120 0.240	0.120 0.240	0.999 0.999			

Values form Table 4 were obtained using: one-way ANOVA statistical analysis used for group comparison (Kruskal–Wallis and Tukey’s test) and t-test used for intragroup comparison of right and left LGN and thalamic volumes. Second row of *p*-values are obtained after Holm–Bonferroni correction.

**Table 5:** Parametric (Pearson’s) and nonparametric (Spearman’s) correlations analysis between LGNs volume and the clinical parameters in studied groups (* *p* < 0.05, ** *p* < 0.01, *** *p* < 0.001).

	Control Group		Early Glaucoma		Advanced Glaucoma		Glaucoma (Early and Advanced)	
	Pearson’s r	Spearman’s R	Pearson’s r	Spearman’s R	Pearson’s r	Spearman’s R	Pearson’s r	Spearman’s R
**LGN volume vs. Age**								
**R**	0.07	0.07	–0.21	–0.20	–0.07	–0.17	–0.30	–0.44
**L**	–0.41	–0.55	–0.11	–0.14	–0.13	–0.17	–0.25	–0.42
**LGN volume vs. MD**								
**MD–LGN (R & L) corresponding eye**	–	–	−0.21	−0.25	−0.14	−0.13	−0.56 **	−0.58 ***
**MD–LGN (R & L) opposite eye**	–	–	−0.31	−0.32	−0.13	−0.13	−0.58 ***	−0.61 ***
**LGN volume vs. mean RNFL**								
**RNFL–LGN (R & L) corresponding eye**	0.51	0.41	**0.57** **	**0.53** *	0.18	0.10	**0.62** ***	**0.52** **
**RNFL–LGN (R & L) opposite eye**	**0.58** *	**0.58** *	**0.58** **	**0.53** *	0.30	0.17	**0.64** ***	**0.56** ***
**LGN volume vs. VFI**								
**VFI- LGN (R & L) corresponding eye**	–0.25	–0.24	0.11	0.04	0.38	0.45	**0.56** **	**0.53** **
**VFI- LGN (R & L) opposite eye**	–0.25	–0.24	0.19	0.16	0.29	0.35	**0.56** **	**0.58** **
**LGN volume vs. quadrant RNFL**								
**LGN–Inferior (R & L) corresponding eye**	0.43	0.23	**0.47** *	0.44	–0.22	–0.31	**0.53** **	**0.40** *
**LGN–Inferior (R & L) opposite eye**	0.49	0.44	**0.55** *	**0.51** *	–0.15	–0.31	**0.58** ***	**0.47** **
**LGN–Superior (R & L) corresponding eye**	0.23	0.31	**0.50** *	**0.50** *	**0.51** *	0.33	**0.62** ***	**0.61** ***
**LGN–Superior (R & L) opposite eye**	0.50	0.45	**0.47** *	0.37	**0.62** *	0.37	**0.63** ***	**0.63** ***
**LGN–Nasal (R & L) corresponding eye**	0.33	0.40	0.01	–0.07	–0.06	–0.08	0.15	0.13
**LGN–Nasal (R & L) opposite eye**	0.24	0.28	–0.08	–0.13	–0.01	–0.17	0.12	0.12
**LGN–Temporal (R & L) corresponding eye**	**0.59** *	0.50	**0.66** **	**0.63** **	0.06	0.14	**0.42** *	**0.51** **
**LGN–Temporal (R & L) opposite eye**	0.48	**0.62** *	**0.68** **	**0.65** **	0.17	0.26	**0.46** *	**0.54** ***
